# Fibrinogen Oxidation and Thrombosis: Shaping Structure and Function

**DOI:** 10.3390/antiox14040390

**Published:** 2025-03-26

**Authors:** Francesca Nencini, Elvira Giurranna, Serena Borghi, Niccolò Taddei, Claudia Fiorillo, Matteo Becatti

**Affiliations:** Department of Experimental and Clinical Biomedical Sciences “Mario Serio”, University of Firenze, Viale Morgagni 50, 50134 Firenze, Italy; francesca.nencini@unifi.it (F.N.); elvira.giurranna@unifi.it (E.G.); serena.borghi@unifi.it (S.B.); niccolo.taddei@unifi.it (N.T.); claudia.fiorillo@unifi.it (C.F.)

**Keywords:** fibrinogen, oxidation, oxidative stress, post-translational modifications, thrombosis

## Abstract

Fibrinogen, a pivotal plasma glycoprotein, plays an essential role in hemostasis by serving as the precursor to fibrin, which forms the structural framework of blood clots. Beyond coagulation, fibrinogen influences immune responses, inflammation, and tissue repair. Oxidative stress, characterized by an imbalance between reactive oxygen species (ROS) and antioxidants, induces fibrinogen oxidation, significantly altering its structure and function. This narrative review synthesizes findings from *in vitro*, *ex vivo*, and clinical studies, emphasizing the impact of fibrinogen oxidation on clot formation, architecture, and degradation. Oxidative modifications result in denser fibrin clots with thinner fibers, reduced permeability, and heightened resistance to fibrinolysis. These structural changes exacerbate prothrombotic conditions in cardiovascular diseases, diabetes, chronic inflammatory disorders and cancer. In contrast, “low-dose” oxidative stress may elicit protective adaptations in fibrinogen, preserving its function. The review also highlights discrepancies in experimental findings due to variability in oxidation protocols and patient conditions. Understanding the interplay between oxidation and fibrinogen function could unveil therapeutic strategies targeting oxidative stress. Antioxidant therapies or selective inhibitors of detrimental oxidation hold potential for mitigating thrombotic risks. However, further research is essential to pinpoint specific fibrinogen oxidation sites, clarify their roles in clot dynamics, and bridge the gap between basic research and clinical practice.

## 1. Introduction

Fibrinogen is a key plasma glycoprotein synthesized mainly in hepatocytes, circulating in the bloodstream at concentrations ranging from 1.5 to 4 g/L with a half-life of 3 to 5 days. As the precursor to fibrin, it plays a pivotal role in coagulation, where the thrombin-mediated cleavage of fibrinogen results in the formation of a fibrin network that stabilizes blood clots, preventing hemorrhage and promoting wound healing. Fibrinogen is composed of three non-identical peptide chains—Aα, Bβ, and γ—containing 610, 461, and 411 amino acids, with molecular weights of 67.5, 55, and 46.5 kDa, respectively. These chains are linked by 29 disulfide bonds. Coagulation is triggered by the serine protease thrombin, and fibrin is further stabilized by covalent crosslinking through the Transglutaminase Factor XIII (FXIII), which enhances clot stability, elasticity, and resistance to fibrinolysis. FXIII also links α2-antiplasmin and plasminogen activator inhibitors to fibrin, contributing to clot resistance against enzymatic degradation [1,2,3,4].

Beyond hemostasis, fibrinogen serves multiple physiological roles, influencing immune responses, inflammation, and tissue repair [5,6]. It acts as a bridge between platelets and promotes the correct spatial arrangement of erythrocytes and macrophages around a wound, thereby facilitating tissue regeneration [3,7]. Additionally, fibrin(ogen) is implicated in defense mechanisms against microbial invasion, forming protective barriers that trap pathogens and recruit immune cells [8,9,10].

Fibrinogen plays a crucial role in tissue repair by forming a provisional extracellular matrix that supports cell adhesion, migration, and activation [11,12]. Converted into fibrin by thrombin, it creates a clot that acts as a scaffold for platelets, immune cells, fibroblasts, and endothelial cells, promoting angiogenesis, fibroblast proliferation, and extracellular matrix deposition [13]. Oxidized fibrinogen significantly alters these processes by modifying platelet aggregation, clot formation, and erythrocyte deformability, contributing to pro-inflammatory and atherosclerotic conditions [14,15]. Platelets are the first responders in vascular injury, and fibrinogen serves as a key mediator in platelet aggregation [12,14]. When platelets are activated, they expose integrin αIIbβ3, which binds fibrinogen and facilitates platelet crosslinking to form a stable clot. The presence of oxidized fibrinogen alters platelet function by enhancing ADP-induced aggregation while impairing ristocetin- and collagen-induced responses [15]. Additionally, oxidized fibrinogen increases the expression of adhesion molecules in endothelial cells (P-selectin, ICAM-1), facilitating leukocyte recruitment and sustaining chronic inflammation, which can impair healing and promote fibrosis or chronic wounds [15,16,17,18,19]. It also enhances reactive oxygen species (ROS) production by leukocytes, exacerbating oxidative stress and promoting persistent inflammatory conditions [20,21,22]. Fibroblasts, essential for extracellular matrix synthesis, are also negatively affected: oxidation alters fibrinogen’s structural properties, reducing its susceptibility to fibrinolysis and impairing fibroblast adhesion and migration, leading to defective tissue remodeling [16,23,24,25,26,27]. In vascular smooth muscle cells, fibrinogen regulates adhesion, proliferation, and migration, processes essential for vascular repair, but its excessive accumulation can promote atherosclerosis progression and intimal thickening, increasing the risk of vascular occlusion [28,29,30]. Qualitative and quantitative alterations in fibrinogen disrupt the delicate balance between inflammation, cell recruitment, and extracellular matrix remodeling, leading to inefficient or pathological tissue repair.

Fibrinogen dysregulation can lead to various pathological conditions. Elevated fibrinogen levels have been associated with an increased risk of cardiovascular (CV) diseases, including coronary artery disease and stroke, due to the formation of denser fibrin clots that are more resistant to lysis, promoting thrombosis [31,32,33]. Conversely, inherited or acquired deficiencies, such as afibrinogenemia and hypofibrinogenemia, can result in spontaneous bleeding disorders [34,35]. Structural abnormalities, like dysfibrinogenemia, further impair fibrin polymerization and clot stability, contributing to a range of bleeding or thrombotic complications [3,34,36].

Fibrinogen also plays a significant role in cancer progression [37]. Increased vascular permeability, driven by factors like VEGF, leads to fibrinogen extravasation and the formation of a fibrin matrix within the tumor microenvironment. This matrix acts as a scaffold for tumor migration, fibroblast recruitment, and angiogenesis. Fibrinogen deposition in the extracellular matrix supports tumor progression by binding growth factors, enhancing adhesion, and promoting inflammation [37,38,39,40,41,42,43,44]. Over time, the fibrin matrix remodels into a collagen-rich stroma, driving desmoplasia and therapy resistance [39,45]. Additionally, fibrinogen interacts with integrins and MMPs, influencing invasion and stromal remodeling, highlighting its role in linking vascular leakage to tumor fibrosis [37,38,39,45]. Although hepatocytes are the primary source of plasma fibrinogen, various tumor cell lines, including lung and breast adenocarcinomas, have been reported to synthesize, secrete, and deposit fibrinogen into the extracellular matrix [38,46]. Similar production has been observed in cervical and intestinal adenocarcinomas, with gene expression confirmed in breast and lung tumors [47,48,49,50]. Fibrinogen deposition is also present in mesothelioma, colon cancer, lymphoma, and breast tumor stroma [51]. This suggests that tumor cells may manipulate their microenvironment by producing fibrinogen to support primary tumor growth, while plasma fibrinogen plays a key role in tumor dissemination and metastasis [51,52].

Prothrombotic fibrin clot features have been reported in patients with hematological cancers and in patients with active solid cancer, including lung and gastrointestinal tumor [40,42]. All parameters of clot formation and lysis emerged as predictors of arterial thrombogenesis and mortality in the oncologic setting, and may help to identify cancer patient subgroups at high risk for these events [53]. In addition, fibrinogen contributes to neurological disorders such as Alzheimer’s disease and multiple sclerosis, where its extravasation into the central nervous system (CNS) exacerbates neuro-inflammation and neurodegeneration [3,54,55].

Fibrinogen is thus a multifaceted protein, whose quantitative and qualitative changes and the resulting different structures and resistances of fibrin clots to mechanical stress or enzymatic breakage are influenced by environmental and genetic factors.

Congenital fibrinogen disorders, such as afibrinogenemia, hypofibrinogenemia, and dysfibrinogenemia, are caused by various genetic mutations in the fibrinogen genes (FGA, FGB, and FGG) [56]. Polymorphisms, such as the γ’ variant in the fibrinogen γ chain or Thr312Ala substitution in the fibrinogen α chain, also affect fibrinogen levels and the architecture of fibrin clots [57,58]. Additionally, polymorphisms in Factor XIII, as well as variants of thrombin and prothrombin, can have a significant impact on fibrin structure [59,60,61]. Environmental influences are often modifiable and interact with genetic predispositions to shape fibrinogen levels and fibrin clot properties. Factors such as smoking, diabetes and hyperglycemia, hyperhomocysteinemia, inflammation, oxidative stress, and medications significantly influence fibrinogen heterogeneity and clot structure [5,61,62,63,64,65,66].

Also, fibrinogen post-translational modifications (PTMs), such as phosphorylation, glycosylation, oxidation, and nitration, further modulate its structure and function, influencing clot formation, architecture, and stability [67,68,69,70,71]. PTMs naturally occur in the human body, where they regulate physiological processes such as cell differentiation and gene expression. However, when the balance between oxidant and antioxidant species is lost, resulting in high circulating levels of reactive species or free radicals, these modifications can affect major macromolecules, particularly proteins, causing structural damage and, consequently, impairing their function [61,71,72]. Among the blood plasma proteins, fibrinogen is known to be the most frequent target of PTMs [73], and among PTMs, oxidation has been extensively studied.

Numerous experimental evidences link inherited fibrinogen variants to thrombotic or bleeding phenotypes [61,74,75,76,77,78,79,80], and mutations and polymorphisms of fibrinogen probably affect PTM sites. However, phosphorylation and glycosylation by top-down mass spectrometry has been performed in only one study [81], a simultaneous analysis of fibrinogen subunits for sequence polymorphisms. The results showed that the Thr312Ala mutation (rs6050) in the Aα chain affects phosphorylation levels, with homozygous carriers showing a different phosphorylation pattern compared to heterozygous or non-carriers. This suggests that genetic variations may directly or indirectly modulate PTM susceptibility, further impacting fibrinogen function.

This review compiles studies selected for their relevance, scientific impact, and contribution to understanding the effects of oxidation on fibrinogen and clot formation. A comprehensive literature search was conducted using databases such as PubMed, Scopus, and Web of Science, with a focus on recent publications that provided novel data or perspectives beyond existing reviews. By synthesizing findings from *in vitro*, *ex vivo*, and clinical research, this narrative review highlights key advancements and ongoing discussions on how oxidation alters fibrinogen’s structure and function, with particular emphasis on its role in coagulation and fibrin clot mechanics.

## 2. Mechanisms of Fibrinogen Oxidation

Oxidation occurs when reactive oxygen species (ROS) are produced excessively and not neutralized by antioxidants. Molecular oxygen itself is biradical, with two unpaired electrons. Key primary oxygen-derived reactive compounds include superoxide (O_2_^•−^) and the highly reactive hydroxyl radical (^•^OH), which is generated from O_2_^•−^ and hydrogen peroxide (H_2_O_2_) in the presence of metal ions via the Fenton reaction. The hydroxyl radical has an extremely short half-life of approximately 10^−9^ seconds. Less reactive ROS include the alkoxyl radical (RO^•^) and peroxyl radical (ROO^•^), both of which play crucial roles as intermediates in lipid peroxidation chain reactions. Nitric oxide (NO) is a relatively slow-reacting molecule with significant importance as a signaling molecule. However, it reacts rapidly with superoxide, producing peroxynitrite (ONOO^−^), which can decompose spontaneously into nitrogen dioxide (NO_2_) and hydroxyl radicals [64].

ROS are by-products of normal cellular metabolism, primarily generated in mitochondria during aerobic respiration. Other enzymatic sources include NADPH oxidases, xanthine oxidase, and nitric oxide synthases. Also, ionizing radiation and pollutants contribute to ROS formation [82,83]. At controlled levels, ROS play crucial roles in cell signaling, in immune response, facilitating microbial killing by neutrophils and macrophages, and in apoptosis [84,85]. Under normal conditions, ROS are neutralized by endogenous antioxidants like superoxide dismutase (SOD), catalase, and glutathione peroxidase. However, external stressors or metabolic dysfunctions can overwhelm these defenses, leading to oxidative stress [86].

Excessive ROS can damage cellular components by inducing lipid peroxidation, which compromises membrane integrity; alters protein structures, resulting in loss of function or aggregation; and causes gene mutations and genomic instability [62,64].

**Figure 1:**
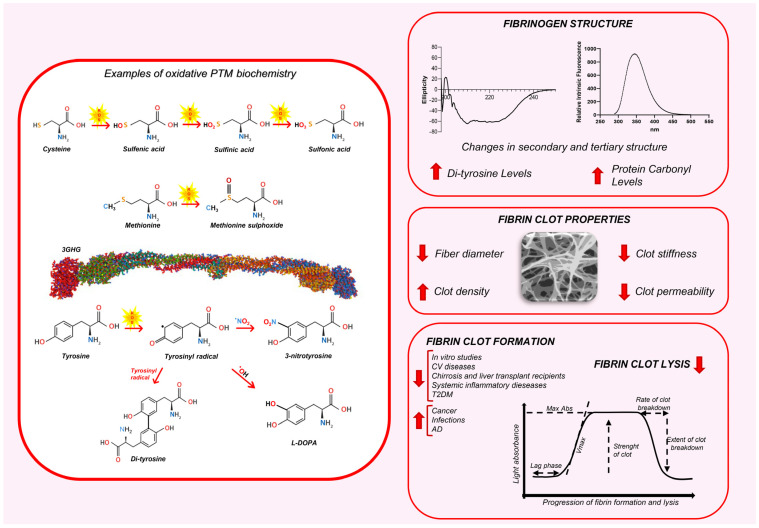
The impact of oxidative PTMs on fibrinogen structure and fibrin clot properties. The left panel illustrates some examples of oxidative PTM biochemistry. The cysteine, methionine, and Tyrosine with the biochemical changes are depicted. The right panel displays the effects of oxidative PTMs on fibrinogen structure, fibrin clot architecture, and clot dynamics. AD: Alzheimer’s disease; CV: cardiovascular; T2DM: type 2 diabetes mellitus.

The consequences of protein oxidation, a primary target for oxidants, depend on the nature of the reactive species involved. Highly reactive species cause widespread damage across side chains and backbones, while less reactive species exhibit greater selectivity for specific residues [87]. This diversity in reaction sites leads to a broad spectrum of PTMs, altering protein composition, folding, net charge, and hydrophobicity or hydrophilicity [88]. These changes significantly impact the protein functions [87,89]. Sulfur-containing amino acids (AAs), such as methionine and cysteine, are highly susceptible to oxidation due to the low oxidative potential of sulfur, making them the AAs most vulnerable to ROS. Cysteine oxidation can result in the formation of sulfenic, sulfinic, and sulfonic acids. Irreversible modifications, such as sulfonic acid formation, indicate a strong oxidative environment. Methionine is oxidized to methionine sulfoxide (Met-S-SO and Met-R-SO), with potentially protective or harmful effects on protein function (Figure 1) [87,89].

Tyrosine, Tryptophan, Histidine, and Phenylalanine are also relatively prone to oxidation, attributed to the high electron density in their aromatic rings. Tyrosine oxidation leads to multiple products such as 3-nitrotyrosine, l-3,4-dihydroxylphenylalanine (l-DOPA), and 3,3′-dityrosine [87] (Figure 1). In contrast, the oxidation of lysine, arginine, proline, threonine, and asparagine is comparatively rare, often catalyzed by metals or enzymes [87]. These modifications are largely irreversible under biologically relevant conditions and, if observed, could suggest the presence of high levels of oxidative stress [90,91].

Compared with other plasma proteins, fibrinogen is more susceptible to oxidative modifications [73,92]. The impact of oxidation on fibrinogen behavior varies across experiments, largely influenced by experimental conditions. In *in vitro* studies, fibrinogen oxidation demonstrated significant variability based on the type and dose of the oxidant. For instance, Martinez et al. [23] described 53 oxidation sites where fibrinogen was treated with hypochlorite, 19 oxidation sites with myeloperoxidase (MPO), and 9 sites where fibrinogen was nitrated with 3-morpholinosydnonimine (SIN-1). Eight years later, Sovova et al. described 113 oxidation sites (44 in the Aα chain, 40 in the Bβ chain, and 29 in the γ chain) by analyzing several studies using ozone or HOCl to induce oxidative modifications in a dose-dependent manner [90]. In our recent review, we reported 143 known sites of fibrinogen oxidation (50 in the Aα chain, 56 in the Bβ chain, and 37 in the γ chain), evaluating reviews, original papers, and protein databases [71].

The effects of oxidation on fibrinogen structure and function have been evaluated in numerous *in vitro* and *ex vivo* studies (Table 1 and Table 2), but only a limited number of investigations have focused on specific modifications at individual sites within the molecule. To date, only seven oxidative PTMs at various fibrinogen sites (AαM91, AαM476, BβH16, BβM190, BβM305, BβM367, γM78) have been described in the literature as influencing alterations in clot formation, dissolution, and overall clot properties [23,72,90,93,94,95,96,97,98,99]. These site-specific oxidations decrease the rate of polymerization and fibrinolysis and result in more dense fibrin clots with thinner fibers.

## 3. Impact of Oxidation on Fibrinogen Structure

Oxidation at various sites within the fibrinogen molecule can lead to significant structural changes, impacting its functional properties. Therefore, analyzing these structural alterations is essential in order to understand the potential biological effects of oxidative PTMs. Several studies have investigated fibrinogen structural changes using circular dichroism (CD) spectroscopy [100,101,102,103,104,105,106,125,129,131,132,155], which is sensitive to alterations in proteins’ alpha-helical backbones, and fluorescence spectroscopy, which provides insights into changes in tertiary structure. The formation of carbonyl groups and specific amino acid modifications, such as the conversion of Tyrosine to dityrosine, have also been evaluated [92,100,102,106,107,108,132] (Table 1 and Table 2). Fibrinogen oxidation results in important changes in the secondary structure of the molecule. In fact, for the CD spectrum of fibrinogen, the shape of the negative valley is closely related to the molecular conformations of the main chain. The α helix has two negative valleys centered at 207 and 221 nm, respectively; the β sheet and β turn present negative valleys around 217 nm and positive valleys around 200–205 nm, respectively; while the random coil has a positive valley around 215–218 nm [102]. The measurement of ellipticity (θ) is widely used to monitor these conformational changes in protein. Oxidized fibrinogen displays an altered circular dichroism spectrum. A decrease in α-helical content was observed *in vitro* and in patients with myocardial infarction (MI) [105], Behçet’s syndrome [129], Giant Cell Arteritis (GCA) [106], endometriosis, cirrhosis, and transplant recipients [102,103,105,106,131,132,156]. On the contrary, an increase in α-helix and β-turn content along with a significant decrease in β-sheet content was observed in fibrinogen incubated with H_2_O_2_ and H_2_O_2_-Fe_3_O_4_ [100,101]. These were used as ROS to study the effects of oxidative stress on the structure and polymerization behavior of fibrinogen molecules [100,101]. In Lau et al. [104], the far-UV CD spectra of the control and the oxidized fibrinogen solutions (with increasing HOCl concentrations) showed similar characteristics of α-helical structure, suggesting that the HOCl oxidation of fibrinogen did not alter its overall α-helical backbone structure. Fibrinogen from patients with end-stage renal disease (ESRD), a condition accompanied by increased inflammation and oxidative stress, showed no changes in the secondary structure with respect to healthy controls [125].

In the protein molecules, three types of amino acid residues including Tryptophan (Trp), Tyrosine (Tyr), and Phenylalanine (Phe) can emit visible fluorescence when excited by UV light. Due to the chromophore difference in their side chains, Trp, Tyr, and Phe residues can emit different fluorescence excitation and emission spectra. Among them, the fluorescence intensity originating from Trp residues is the maximum, and these residues are sensitive to the changes in the microenvironment and are often used as endogenous probes to study protein tertiary structures [157,158]. The analysis of intrinsic fluorescence spectra revealed a reduction in the peak for fibrinogen oxidized *in vitro* and fibrinogen purified from patients with endometriosis, cirrhosis, and liver transplant recipients [100,101,102,109,131,132,156].

ROS are involved in protein carbonylation that represents the most common nonenzymatic PTM [87]. Carbonyl groups can be introduced into proteins by an oxidative (direct) or nonoxidative (indirect) mechanism, which appears not to be random and leads to an increase in the overall cellular load with protein carbonyls. Elevated protein carbonyl (PC) levels are considered an early biomarker of exposure to ROS [159]. A dose-dependent increase in the content of carbonyl groups in oxidized fibrinogen was observed in all the *in vitro* studies [92,105,110,111,112], regardless of the oxidizing agent used (Table 1).

Among the *ex vivo* studies (Table 2), carbonyl content has been assessed in various diseases, such as cardiovascular diseases [118,119,120], diabetes [127], cirrhosis [102,131,133], liver transplantation [132], ESRD [125], and systemic inflammatory diseases characterized by thrombotic tendency (Behçet’s disease and GCA) [106,129], as well as multiple myeloma [135] and neurodegenerative diseases [151]. In all studies, a marked carbonylation of fibrinogen was observed in the patients compared to healthy controls. The extent of carbonylation is often associated with disease severity and the formation of clots with a procoagulant structure, characterized by reduced permeability and increased resistance to lysis.

The interaction of oxidizing agents with Tyrosine residues within the fibrinogen polypeptide chain leads to the formation of tyrosyl radicals [87]. These radicals can couple through the *ortho–ortho* crosslinking of their hydroxyl groups, resulting in the formation of dityrosine (diTyr) bridges. These bridges, connecting two or more polypeptide chains, facilitate the creation of high-molecular-weight aggregates [160]. These aggregates are thought to possess not only greater masses but also distinct biological properties [161]. Consequently, dityrosine production has been linked to the formation of a denser fibrin network and modifications in clot structure. These observations were reported in several *in vitro* and *ex vivo* studies [92,100,102,106,107,108,132].

Numerous observations also show that fibrinogen oxidation induces chemical modifications of highly susceptible methionine residues, such as AαM476, which is the most preferentially oxidized methionine in HOCl oxidation experiments [96,97,98,108,113]. This residue is the first in a second β-sheet hairpin structure within the N-terminal subdomain of the αC domain. The instability of this region impairs the lateral aggregation of protofibrils, resulting in smaller fibers and increased fiber density [97,98,99,134]. Recently, Yurina et al. [96] showed that a low concentration of HOCl/OCl (10 μM) as an oxidizing agent did not affect either the fibrin network structure or the kinetics of the fibrinogen-to-fibrin conversion or fibrinolysis. They also observed that, in this experimental system, some methionine residues—AαMet476, AαMet517, AαMet584, BβMet367, γMet264, and γMet94—underwent transformation into methionine sulfoxide/sulfone, acting as scavengers of ROS and playing a crucial antioxidant role, which disappears as the dose of the oxidizing agent increases. This is probably an evolution process of protein structure adaptation in response to oxidative stress, observed in fibrinogen and in other molecules [162,163,164].

## 4. Impact of Oxidation on Fibrin Clot Architecture

The association of the structural properties of fibrin fibers and the network with clinical pathologies suggests that the characteristics of the fibrin clot architecture are crucial in maintaining physiological hemostasis and are therefore a good predictive marker of coagulation dysfunction [31,165]. The impact of oxidative PTMs on clot properties can be assessed by measuring fibrin fiber diameter, clot stiffness, clot permeability, and clot density.

*In vitro* studies (Table 1) employing various oxidation protocols (e.g., irradiation, photooxidation, ascorbate/FeCl3, peroxynitrite, HOCl, glycolaldehyde, hydrogen peroxide) consistently showed a reduction in fibrin fiber diameters [96,97,99,100,101,104,108,110,112,113]. However, one *in vitro* study presents conflicting results [111]. Roselfeld et al. showed that fibrin formed from fibrinogen ozonation was characterized by a rougher structure and higher average fiber mass/length ratio compared with native fibrin [111]. Different methods used to generate specific ROS aim to replicate the alterations in protein molecules observed during oxidative (patho)physiological reactions occurring *ex vivo* [166]. However, *in vitro* modification with specific reagents is only an approximation of real conditions, and the use of different oxidizing agents and varying concentrations can often produce confounding results [167,168]. Identifying PTM sites and the precise mechanisms underlying fibrin formation damage remains highly challenging.

Among *ex vivo* studies (Table 2), the fibrin fiber diameter has been assessed in various conditions. In liver transplantation, in Behçet’s disease, and GCA, as well as in cardiovascular diseases and lung cancer, fibrin fibers have smaller diameters [1,102,105,106,119,121,126,129,130,132,136,152].

Paton et al. [118] showed that, in post-MI patients (within 24–96 h of the event), changes in the high carbonyl plasma reflect a faster rate of the lateral aggregation of small oligomers to form fibrin polymers that comprise thicker, more loosely woven fibers. *Ex vivo*, this could be translated into a tendency to clot faster and form more fragile clots. In contrast, our group observed thinner fibers in patients with post-acute MI (6 months after the event). This discrepancy could be attributed to differences in the patient cohorts enrolled in the studies.

In the study by Hugenholtz et al. [133], the fiber thickness in cirrhosis patients was found to be largely comparable to that of healthy volunteers. In contrast, our group [102], by super-resolution microscopy, showed a significant reduction in fibrin fiber diameter in cirrhotic patients compared to controls, with this difference becoming more pronounced as the disease progressed. In ESRD, fibrin from plasma samples of patients on peritoneal dialysis (PD) exhibited fiber thickness like that of the control group, as observed in SEM images [125]. The authors suggest that other factors, besides fibrinogen oxidation, contribute to fibrinogen behavior in patients with ESRD, especially considering that both bleeding and thrombosis were recorded in such condition [125].

Fibrin is a viscoelastic polymer subjected to shear stress within blood vessels due to blood flow. Its elasticity, or stiffness, refers to its ability to undergo reversible mechanical deformation, while its viscosity, or plasticity, involves slow, irreversible deformation [169,170]. The mechanical properties of fibrin are critical to its function. Clot stiffness has been extensively studied. Both *in vitro* and *ex vivo* research has shown that oxidized plasma fibrin gels exhibit structural heterogeneity and reduced gel stiffness [1,97,99,102,104,106,107,108,114,122,130,132,134] (Table 1 and Table 2).

In the study by Ullah et al. [137], the authors examine differences in blood viscoelasticity and plasma protein levels among peritoneal cancer patients (stage IV with metastasis) of different origin (colic, pseudomyxoma, gastric, rectal, ovarian, and others), individuals with other diseases, and healthy controls. Their findings demonstrate that clots with higher elasticity, particularly those from cancer patients, contain thicker, more condensed fibers compared to clots with lower elasticity. These results indicate that cancer patients exhibit a hypercoagulable state, which may influence their hemostatic balance and increase thrombotic risk.

Fibrin clot permeability, which reflects the average pore size between fibrin fibers, is currently the most used parameter for assessing fibrin clot structure in various disease conditions [171,172]. Reduced fibrin clot permeability has been linked to recurrent thrombotic events and has been observed not only in patients with thromboembolism but also in a range of conditions associated with an elevated risk of thromboembolic events, including cancer, diabetes, cirrhosis, and inflammatory diseases [1,102,106,119,121,126,127,128,132,133,136,138,139,140,141]. Reduced fibrin clot permeability was observed in several *in vitro* studies (Table 1), where fibrinogen was oxidized with HOCl/^−^OCl, a strong oxidizing agent responsible for the killing action of neutrophils against a variety of pathogens [173]. The concentrations of HOCl/^−^OCl used varied from 25 to 300 μM, since *ex vivo* levels are estimated to be able to reach mM ranges. The recent results of Yurina et al. [96] provided evidence that fibrinogen treated with a low dose of HOCl/^−^OCl (10 μM) undergoes dose-dependent chemical modifications without altering the structure of the fibrin network.

Advanced lung cancer, digestive tract cancer, and hematological malignancy (multiple myeloma and essential thrombocythemia), are associated with impaired plasma clot characteristics, with a tendency to form less permeable fibrin clots [136,138,139,140]. In the study by Mrozinska et al. [141], the authors hypothesized that prothrombotic plasma fibrin clot properties, evaluated after an unprovoked thrombosis, could serve as predictors of occult malignancy. Their findings revealed that specific prothrombotic features, namely, lower Ks and prolonged clot lysis time, measured 2 to 8 months following an unprovoked venous thromboembolism event, were predictive of a cancer diagnosis within three years in patients under 70 years of age [141]. These results could have practical implications for cancer screening strategies. Also, in type 2 diabetes mellitus (T2DM), there is an association with a prothrombotic state, including increased thrombin generation and platelet hyperactivity as well as endothelial dysfunction [174,175]. A number of studies have demonstrated that the structure and function of fibrin clots are unfavorably altered in subjects with T2DM [176,177,178]; in particular, enhanced oxidative stress adversely affects plasma fibrin clot permeability, regardless of disease duration and glycemia control [127,128]. Clot permeability was also significantly reduced in patients with cirrhosis [102,133], in liver transplant recipients [132], and in hemodialysis patients [126], as well as in patients with arterial atherothrombotic disorders [1,106,119].

Clot density, defined by the compactness and organization of the fibrin network, is another critical parameter of hemostatic balance and is often heightened in prothrombotic conditions, indicating the formation of a tightly packed and less permeable fibrin matrix [179]. *In vitro* studies (Table 1) consistently demonstrate an increased clot density [96,97,99,100,101,103,108,112,113], except for Roitman’s work in 2004 [114]. When fibrinogen was oxidatively modified by UV irradiation and added to tubes containing 10 mL of citrated blood, the oxidized fibrinogen solution (10%) moderately activated the intrinsic coagulation pathway while inhibiting the extrinsic pathway. Fibrinogen with a 20% oxidation degree inhibited both the extrinsic and intrinsic pathways, leading to reduced clot rigidity and density and impairing normal fibrin clot formation [114]. *Ex vivo* studies (Table 2) have shown that clots of their patients appeared to be more confluent and tightly packed (denser) than the looser fibrin networks of control donors [102,106,119,123,130,132,136,140,142,143,152]. Fibrin fiber network analysis thus suggests that patients displayed a matted clot structure with fewer open spaces visible compared to control clots. In patients with cancer (colorectal, breast, and lung) [136,142,143] and multiple myeloma [140], as well as in patients with cirrhosis [102], liver transplant recipients [132], individuals with systemic inflammatory [106,129,130], and cardiovascular diseases [119,121,123], an increased tendency to develop larger and denser clots compared to controls has been observed.

Similarly, in the case report by Ceznerová [121], which describes the first documented instance of hypofibrinogenemia associated with thrombosis—fibrinogen Predmerice—linked to mutations in the N-terminal region of the γ chain, scanning electron microscopy revealed distinct morphological differences between the patient’s fibrin network and that of a healthy control. The fibrin fibers in the patient exhibited a denser spatial arrangement, with smaller pores composed of shorter fibers. Additionally, they appeared more branched, compact, and structurally rigid [121]. On the other hand, we have already discussed the study by Paton et al. [118] which showed that in post-MI patients there is a faster lateral aggregation of small oligomers to form fibrin polymers, leading to thicker and looser fibers which result in more fragile and less dense clots. The study by Zamolodchikov et al. [152] is the only study that investigated the properties of fibrin clots in Alzheimer’s disease (AD). AD is characterized by elevated levels of β-amyloid peptide (Aβ) in the brain parenchyma and cerebral blood vessels. This *in vitro* study demonstrated that Aβ binding to fibrin promotes the formation of a denser fibrin network with thinner fibers, which hinders plasmin(ogen)’s access to fibrin and delays fibrinolysis [152].

## 5. Impact of Oxidation on Clot Formation

Fibrinogen is a complex glycoprotein composed of distinct structural regions, each with specific functions. The central E region contains thrombin cleavage sites and connects to two D regions via coiled-coil domains, which provide elasticity. The D regions house binding holes essential for fibrin polymerization. While the Bβ and γ chains terminate in the D region, the Aα chain extends further, forming a flexible αC region crucial for fibrin fiber assembly [31]. Thrombin cleaves fibrinopeptides A and B from the Aα and Bβ chains, exposing binding sites in the E region that interact with complementary holes in the D regions. This initiates fibrin polymerization into protofibrils. The cleavage of fibrinopeptide B further promotes αC interactions, leading to the lateral aggregation of protofibrils into fibrin fibers. Factor XIII, activated by thrombin, stabilizes fibrin through the covalent crosslinking of the γ and α chains, enhancing clot durability and resistance to fibrinolysis [6,165,180,181,182,183].

Oxidative PTMs of fibrinogen can markedly influence clot formation kinetics, which can be assessed through the following key parameters: (i) thrombin-catalyzed fibrin polymerization, measuring the conversion of fibrinogen to fibrin and determining clotting time or aggregation rate; (ii) maximum velocity (Vmax), reflecting the rate of lateral protofibril association; (iii) lag phase, indicating the delay before fibril aggregation initiates; and (iv) maximum turbidity or absorbance (MaxAbs), representing the final clot structure in terms of fibrin fiber thickness and protofibril density [102]. *In vitro* studies consistently show that fibrinogen oxidation impairs its conversion to fibrin relative to its nonoxidized counterpart (Table 1). A prolonged lag phase is a widely observed phenomenon across multiple experiments [92,105,106,110,111,115], while both maximum absorbance and maximum velocity, assessed through turbidity assays, exhibit a significant reduction [92,93,96,103,105,106,107,110,112,113,115,116]. However, the effects of oxidation on fibrin clot formation are not entirely uniform. While Torbitz et al. [117] reported an increased polymerization rate, other studies [92,96,97] found no notable differences in fibrinogen-to-fibrin conversion. A possible explanation for this can be found in Nowak and Yurina’s studies [92,96]. While concentrations of 100–1000 μmol of peroxynitrite or 25 μM of HOCl reduce the polymerization rate, in line with the literature, lower concentrations (10 μmol of peroxynitrite and 10 μM of HOCl) do not induce any changes. In the study by Torbitz, the concentrations of HOCl were 10 times higher than the average concentrations used, which could explain its opposite effect on clotting activity. *Ex vivo* observations (Table 2) show more complex and heterogeneous results. Most of them report that in endometriosis, liver transplantation, cirrhosis, Behçet’s disease, and GCA, as well as in cardiovascular diseases, fibrinogen oxidation significantly reduces its conversion to fibrin compared to nonoxidized fibrinogen [1,102,105,106,121,122,129,130,132,133,134,156]. Patients with ESRD on hemodialysis, MI, COVID-19, and different types of cancer have yielded conflicting results [118,126,136,137,142,144,146,153]. For instance, Undas et al. [126] observed a faster fibrin polymerization that could contribute to the progression of atherothrombotic vascular disease in hemodialysis patients. Similarly, Paton et al. showed [118] a higher polymerization rate and increased maximum turbidity in oxidized fibrinogen from MI patients (within 24–96 h of the event). As previously discussed, these results are in contrast with our data [105], where a slower rate of thrombin-catalyzed fibrinogen polymerization in patients with post-acute MI (6 months after the event) was observed. This discrepancy could be attributed to differences in the patient cohorts enrolled in the studies. Okazaki et al. [146] conducted a very interesting study on a cohort of COVID-19 patients. Both mild and severe COVID-19 patients were found to exhibit a hypercoagulable state, as evidenced by a decrease in clotting time (CT) and an increase in Maximum Clot Firmness (MCF) and clot strength at 20 min (A20). This hypercoagulable state manifests early in severe cases and later in mild cases. In both conditions, but particularly in mild COVID-19, there is a tendency for the hypercoagulable state to reverse by the end of the first month after symptom onset [146].

Altered *ex vivo* properties of plasma clot formation have been observed in patients with cancer [53,136,137,142,144]. A prothrombotic state is frequently observed in these patients and contributes to the risks of venous thromboembolism (VTE), arterial thromboembolism (ATE), tumor progression, and death. Compared to controls, cancer patients exhibited increased clot formation potential and elevated levels of biomarkers associated with inflammation and hemostasis, such as C-reactive protein, FVIII, and thrombin generation [53]. These findings strongly support the concept of a shared pathobiology involving inflammation, hypercoagulability, hyperfibrinogenemia, and plasma clot alterations in the context of cancer [53,137,138,142]. This concept does not apply to all cancers. For example, in patients with advanced lung cancer [136], researchers observed a prothrombotic plasma clot phenotype largely driven by smoking and independent from increased plasma fibrinogen or D-dimer or thrombin generation. In Goncalves’ study [143], early disease, treatment-naïve breast cancer (BC) patients presented with visible alterations to fibrin clot structure and statistically significant changes to their fibrinogen structure, but less marked changes to the ultrastructure of their blood cells and no significant viscoelastic changes. The study points out that the coagulation system may react in different ways to the disease, depending on the progression of the disease itself. Patients with more aggressive tumors have less favorably altered fibrin clot properties than lower-grade tumor patients [138,142].

Three studies underscore the multifaceted impact of multiple myeloma (MM) on hemostasis. MM patients show significant variability in fibrin clot formation and polymerization rate [135,140,144]. Undas et al. [140] observed denser, less permeable clots with slower polymerization (longer lag phase and lower final turbidity) due to elevated thrombin levels. Ghansah et al. [144] highlighted a marked reduction in Factor XIII activity, which compromises clot stability. Hypercoagulability was detected in MM and in monoclonal gammopathy of undetermined significance (MGUS), indicating that a disturbed hemostasis balance is already present in the latter benign condition. In contrast, Nowak et al. [135] emphasized oxidative modifications to fibrinogen, leading to altered clot structures with longer lag phases but no significant changes in maximum velocity. These discrepancies likely stem from the heterogeneity of the studied populations, with varying degrees of disease severity, different measurement techniques, and the focus on specific biochemical pathways.

Also, in Alzheimer’s disease, a prothrombotic state is evidenced by increased clot formation and elevated levels of coagulation factors and activated platelets [153,184].

## 6. Impact of Oxidation on Clot Lysis

Fibrinolysis is the mechanism responsible for breaking down fibrin clots to restore normal blood flow. It is driven by plasmin, which is activated from plasminogen and regulated by inhibitors such as TAFI (Thrombin Activatable Fibrinolysis Inhibitor) and α2-antiplasmin. This system is essential for maintaining hemostatic balance.

Oxidative modifications of fibrinogen, triggered by various oxidants, have been shown to impact hemostasis by altering fibrin assembly and the morphology of the fibrin network [23,71]. These changes also influence clots’ susceptibility to fibrinolysis.

Several *in vitro* studies (Table 1) highlight that fibrinogen oxidation leads to a decreased fibrinolytic activity [96,97,104,105,106,107,108,110,114]. Only three studies do not show changes in clot lysis [96,114,116]. In Roitman et al. [114], the influence of oxidized fibrinogen on the blood coagulation system depends on the degree of oxidation. Fibrinogen with a low oxidation level (10%) produces different effects, likely mitigated by the pool of natural antioxidants. In contrast, highly oxidized fibrinogen disrupts all pathways of blood coagulation [114]. A similar conclusion was reached by Yurina et al. [96], who found that under mild and moderate oxidative stress conditions, their study’s results provided evidence of a protective mechanism that helps maintain the structure and function of fibrinogen molecules in the bloodstream.

*Ex vivo* studies (Table 2) also largely agree that oxidative modifications of fibrinogen reduce fibrinolysis, promoting a prothrombotic phenotype [1,53,102,105,106,119,121,123,124,126,127,128,129,130,132,136,137,138,139,140,141,144,145,146,147,148,149,150,152,153,154,156,185,186,187,188].

This is evident from the impaired clot dissolution observed in inflammatory conditions such as Behçet’s disease [129,189,190], GCA [106], and endometriosis [156].

Poor glycemic control, disease duration, and increased oxidation account for the prothrombotic alterations observed in the plasma fibrin clot characteristics of T2DM patients. Increased protein carbonylation leads to significant hypofibrinolytic effects, further emphasizing the link between oxidative stress and prothrombotic changes in this condition [127,128].

Oxidative modifications in patients with acute coronary syndrome, acute ischemic stroke, significant carotid and aortic stenosis, and atrial fibrillation alter fibrinogen functionality, which in turn negatively impacts the efficiency of coagulation and fibrinolysis [1,105,119,121,122,123,124,185].

Some studies have demonstrated that liver transplant recipients face a significantly higher risk of CV disease compared to the general population. This increased risk is primarily attributed to the high prevalence of metabolic syndrome and the use of immunosuppressive medications [191,192,193]. Additionally, chronic low-grade inflammation, which is common following solid organ transplantation, serves as a recognized nontraditional risk factor for the development of CV events [194,195,196]. In a recent paper, our group [132] investigated the role of oxidation-induced fibrinogen modifications after liver transplant, observing that transplanted patients showed systemic oxidative stress associated with fibrinogen alterations. The increased resistance of fibrin to lysis and a correlation between smoking or donor steatosis and the rate of resistance of fibrin to lysis was also observed. These data are in line with literature findings [197,198,199] and with cirrhosis patients’ observations [102]. In contrast, the study by White et al. [134] found that trauma patients with higher levels of fibrinogen Aα-Met476(SO) experienced impaired fibrin polymerization, resulting in weaker clots and increased fibrinolysis after injury. Furthermore, a strong positive correlation between INR and Aα-Met476(SO)% was consistently observed in all trauma groups [134].

Fibrinolysis plays a crucial role in cancer progression, metastasis, and patient prognosis. In cancer, this system often becomes dysregulated, typically resulting in hypofibrinolysis, where fibrin clots persist longer than normal [53,136,137,138,139,140,141,144,145]. This prothrombotic state not only increases the risk of thrombotic events but also supports tumor growth and metastasis by creating a microenvironment favorable to cancer cells [200,201]. Components of the fibrinolytic system, such as plasminogen activators and inhibitors, are frequently overexpressed in aggressive cancers and serve as biomarkers of poor prognosis [44,202], and fibrin itself acts as a scaffold for tumor cells, providing structural support for cell migration and tumor growth [53,136,137,138,139,141,142,145].

Only MM studies highlight distinct differences in fibrinolysis among patients [135,140,144]. Undas et al. [140] show impaired fibrinolysis, with a prolonged clot lysis time and reduced D-dimer release, linked to increased thrombin formation and TAFI activity. Ghansah [144] et al. report enhanced fibrinolysis, indicated by elevated plasmin generation and D-dimer levels, attributed to reduced Factor XIII activity and ongoing clot destabilization. In contrast, Nowak et al. [135] find no significant difference in fibrinolytic markers but note increased oxidative stress, which may indirectly influence clot lysis dynamics. These variations may arise from differences in patient populations, disease stages, and methodologies.

Fibrinolysis dysregulation is a key factor in the pathophysiology of sepsis and COVID-19. In both conditions, impaired fibrinolysis contributes to the formation of fibrin-rich, lysis-resistant clots, which exacerbate thrombotic complications and organ dysfunction [203,204]. In COVID-19, hypercoagulability is driven by fibrinolytic shutdown, characterized by reduced clot degradation [205]. Some studies reveal that patients with severe disease or requiring intensive care unit (ICU) admission show significantly reduced maximum lysis values, indicating impaired fibrinolysis [146,148,150]. This fibrinolytic dysfunction correlates with higher morbidity and mortality, as dense fibrin clots resist degradation despite anticoagulation therapy [206,207]. Additionally, SARS-CoV-2 directly interacts with fibrinogen, promoting the formation of structurally abnormal, pro-inflammatory clots that exacerbate thrombo-inflammation and oxidative stress [146,148,150]. Similarly, in sepsis, fibrinolytic disturbances are pivotal in disseminated intravascular coagulation and organ failure [203,208]. Clot–lysis assays reveal heterogeneous profiles in septic shock patients, including decreased fibrin formation, normal fibrinolysis, and pronounced lysis resistance [149]. The latter is linked to elevated D-dimer levels, prolonged clot lysis times, and impaired thrombin generation, further aggravating coagulopathy. This variability highlights the complex interplay between hypofibrinolysis and hypercoagulability in septic patients [147,149].

In both diseases, the inflammatory milieu exacerbates fibrinolytic dysfunction. Elevated plasminogen activator inhibitor-1 (PAI-1) levels, commonly seen in these patients, inhibit fibrinolysis, leading to microvascular thrombosis and contributing to multi-organ failure. The presence of fibrin in critical sites, such as the lungs and brain, further drives inflammation and tissue damage, linking coagulation abnormalities to broader systemic effects [146,147,148,149,150].

The critical role of fibrinogen is increasingly recognized in neurodegenerative diseases, with hypofibrinolysis also being reported [152,154,209,210,211,212]. An interesting model proposed by Cortes-Canteli et al. [153] attempts to explain the role of fibrinogen in Alzheimer’s disease (AD) pathology. In this model, β-amyloid peptide accumulates in the brain, associating with fibrinogen in the parenchyma, around blood vessels, or within vessels. This creates a prothrombotic environment that promotes clot formation. The presence of Aβ results in abnormal, lysis-resistant clots [153]. Persistent fibrin may obstruct blood flow or trigger inflammation, leading to neuronal damage and dysfunction [55]. Additionally, fibrinogen in the blood vessels of AD patients may trap Aβ, hindering its clearance through the vasculature. This process could exacerbate the formation of cerebral amyloid angiopathy (CAA), reduce blood circulation, and ultimately contribute to cognitive decline [55,153,184,213].

## 7. Conclusions

Fibrin clot formation and lysis are dynamic processes that maintain the delicate balance between preventing bleeding and avoiding thrombosis, with fibrinogen playing a central role. Oxidative modifications have emerged as key regulators of fibrinogen function, influencing clot structure, stability, and resolution.

Fibrinogen oxidation not only affects the biochemical and mechanical properties of fibrin clots, but also modulates fibrinogen interactions with other blood components, endothelial cells, and plasma proteins, underscoring their importance in both physiological and pathological contexts.

Recent research has established a strong link between thromboembolic events and the specific structural features of fibrin clots, often shaped by oxidative PTMs. Oxidation leads to the formation of denser clots with thinner fibers, reducing clot permeability and increasing resistance to fibrinolysis (Figure 1). These changes contribute to prothrombotic conditions associated with cardiovascular disease, chronic inflammation, diabetes, and cancer, potentially shifting fibrinogen toward a thrombosis-prone state.

The potential for therapeutic intervention targeting fibrinogen PTMs is a promising area of research. Antioxidant therapies, for example, could mitigate oxidative stress and reduce harmful modifications, while specific inhibitors could selectively block detrimental PTMs without interfering with beneficial ones. By altering clot properties to favor fibrinolysis, such strategies could reduce thrombus formation and improve outcomes for patients at risk of thromboembolic events. However, these approaches require a detailed understanding of the mechanisms by which PTMs influence fibrinogen’s structure and function. Mass-spectrometry-based proteomics has provided valuable insights into fibrinogen modifications, but the identification of specific PTM sites and their functional implications is still incomplete. Variability in experimental conditions, detection methods, and patient populations complicates the interpretation and comparison of findings.

To establish a clearer connection between PTMs and thrombotic phenotypes, standardized protocols and comprehensive thrombus analysis are crucial. Variability in oxidation studies, due to differences in experimental conditions such as oxidant concentrations and analytical methods, has led to inconsistencies in findings. Standardization should focus on defining optimal oxidation parameters and ensuring reproducible methodologies to improve data reliability. A comprehensive analysis of thrombus composition should integrate structural characterization through high-resolution imaging techniques, proteomics for mapping oxidation sites, and functional assays to assess clotting and fibrinolysis dynamics. Additionally, aligning *in vitro* oxidation models with patient-derived fibrinogen samples will enhance translational relevance. Identifying oxidative biomarkers, such as protein carbonylation and methionine sulfoxidation, could provide valuable insights into thrombotic risk assessment. By combining these approaches, research can bridge the gap between laboratory studies and clinical applications, paving the way for precision medicine strategies in thrombotic disease management. Future investigations should prioritize longitudinal patient studies to monitor fibrinogen oxidation over time and its role in thrombotic disorders.

## Figures and Tables

**Table 1:** Effects of *in vitro* oxidation on fibrinogen function, clot formation, and degradation.

		Fibrinogen Analysis					Clot Analysis				
			Polymerization Kinetics								
Author	Method	Fibrinogen Polymerization	Lag Phase	Max Abs	Vmax	Fibrinogen Structural Alterations	Fiber Diameter	Stiffness	Permeability	Density	Fibrin Lysis
Nowak et al. [92] (2007)	Fibrinogen + 10 μmol peroxynitrite	**=**	**=**	**=**		Dityr-PC					
Nowak et al. [92] (2007)	Fibrinogen + 100–1000 μmol peroxynitrite	**−**	**+**	**−**		Dityr-PC					
Yurina et al. [95] (2019)	Fibrinogen + 50, 500 or 1500 μmol HOCl/mg fibrinogen	**−**		**−**							
Yurina et al. [96] (2024)	Fibrinogen +10 μM HOCl	**=**		**=**						**=**	**=**
Yurina et al. [96] (2024)	Fibrinogen +25 μM HOCl	**−**		**−**			**−**		**−**	**+**	**−**
Weigandt et al. [97] (2012)	Fibrinogen + 50–150 μmol HOCl/g fibrinogen	**=**					**−**	**−**	**−**	**+**	**−**
Pederson et al. [99] (2019)	Met^476^ unoxidized and oxidized αCdomain dimer						**−**	**−**		**+**	
Wang et al. [100] (2016)	Fibrinogen + H_2_O_2_					CD-Dityr-IF	**−**			**+**	
Wang et al. [101] (2018)	Fibrinogen + 0.5 mM H_2_O_2_ + 3 mg/mL Fe_3_O_4_	**−**				CD-IF	**−**			**+**	
Becatti et al. [102] (2020)	Fibrinogen + 1–4 mM AAPH					CD-IF					
Rosenfeld et al. [103] (2021)	Fibrinogen + 25–300 μM HOCl	**−**		**−**		CD	**−**		**−**	**+**	
Lau et al. [104] (2021)	Fibrinogen + 10–150 μmol HOCl/L	**−**				CD	**−**	**−**			**−**
Becatti et al. [105] (2014)	Fibrinogen + 0.01–1 mM AAPH	**−**	**+**	**−**	**−**	PC					**−**
Bettiol et al. [106] (2023)	Fibrinogen + 0.01–1 mM AAPH	**−**	**+**	**−**	**−**						**−**
Tetik et al. [107] (2011)	Fibrinogen + 100 μM Fe^3+^/ascorbate	**−**			**−**	Dityr		**−**			**−**
Misztal et al. [108] (2019)	fibrinogen + 0–1000 μM HOCl from controls					Dityr	**−**	**−**		**+**	**−**
Gligorijević et al. [109] (2020)	Fibrinogen + 10 mM AAPH					IF					
Andrades et al. [110] (2009)	Bovine fibrinogen or human plasma + 1 mM glycolaldehye	**−**	**+**	**−**	**−**	PC	**−**				**−**
Rosenfeld et al. [111] (2009)	Fibrinogen + 200–600 nmol ozone	**−**	**+**			PC	**+**				
Stikarova et al. [112] (2013)	Fibrinogen + 1.25 mM NaOCl	**−**		**−**		PC	**−**			**+**	
Stikarova et al. [112] (2013)	Fibrinogen + 100 μmol SIN-1			**−**		PC	**−**			**+**	
Yurina et al. [113] (2021)	Fibrinogen +25–50–300 μmol hydrogen peroxide	**−**		**−**			**=**		**=**	**=**	
Yurina et al. [113] (2021)	Fibrinogen +25–50–300 μmol HOCl	**−**		**−**			**−**		**−**		
Roitman et al. [114] (2004)	UV irradiation of fibrinogen	**−**						**−**		**−**	**=**
Azizova et al. [115] (2009)	Fibrinogen + 50–500 μmol FeSO_4_ + 10–250 μmol H_2_O_2_	**−**	**+**		**−**						
Piryazev et al. [116] (2009)	Fibrinogen + 50–500 μmol FeSO_4_ H_2_O_2_	**−**		**−**							**=**
Torbitz et al. [117] (2015)	Fibrinogen + 1, 2, 4 mM HOCl	**+**									

The table summarizes findings from multiple *in vitro* studies analyzing the effects of oxidative PTMs on fibrinogen. “+” denotes an increase, “−” denotes a decrease, “=“ denotes no change. PC, protein carbonyls; CD, circular dichroism; IF, intrinsic fluorescence; Dityr, dityrosine crosslinking.

**Table 2:** Effects of *ex vivo* oxidation on fibrinogen function, clot formation, and degradation.

		Fibrinogen Analysis					Clot Analysis				
			Polymerization Kinetics								
Author	Method	Fibrinogen Polymerization	Lag Phase	Max Abs	Vmax	Fibrinogen Structural Alterations	Fiber Diameter	Stiffness	Permeability	Density	Fibrin Lysis
CARDIOVASCULAR DISEASES											
Kaufmanova et al. [1] (2021)	Fibrinogen from patients with arterial atherothrombotic disorders	**−**		**−**			**−**	**−**	**−**		**−**
Becatti et al. [105] (2014)	Fibrinogen from MI patients	**−**	**+**	**−**	**−**	PC-CD	**−**				**−**
Paton et al. [118] (2010)	Fibrinogen from MI patients	**+**	**=**	**+**	**+**	PC	**+**			-	
Błaż et al. [119] (2023)	Plasma from acute ischemic stroke patients					PC	**−**		**−**	**+**	**−**
Nowak et al. [120] (2024)	Plasma from atrial fibrillation patients					PC					
Ceznerova et al. [121] (2022)	Fibrinogen from patient with thrombosis-associated hypofibrinogenemia	**−**		**−**			**−**		**−**	**+**	**−**
Siudut et al. [122] (2022)	Fibrinogen from patients with aortic stenosis			**−**	**−**			**−**			**−**
Słaboszewski et al. [123] (2024)	Plasma from atrial fibrillation patients									**+**	**−**
Sumaya et al. [124] (2018)	Plasma from acute coronary syndrome										**−**
RENAL DISEASES											
Baralic et al. [125] (2020)	Fibrinogen from patients with ESRD (end-stage renal disease)					CD-PC	**=**	**=**	**=**	**=**	
Undas et al. [126] (2008)	Fibrinogen from haemodialysis patients	**+**	**−**		**+**		**−**		**−**		**−**
TYPE 2 DIABETES											
Bryk et al. [127] (2019)	Fibrinogen from type 2 diabetic patients					PC			**−**		**−**
Lados-Krupa et al. [128] (2015)	Fibrinogen from type 2 diabetic patients								**−**		**−**
SYSTEMIC INFLAMMATORY DISEASES											
Becatti et al. [129] (2016)	Fibrinogen from patients with Behçet disease	**−**	**+**	**−**	**−**	PC-CD					**−**
Bettiol et al. [106] (2023)	Fibrinogen from GCA patients	**−**	**+**	**−**	**−**	CD-Dityr-IF	**−**	**−**	**−**	**+**	**−**
Becatti et al. [130] (2019)	Fibrinogen from patients with Behçet disease	**−**	**+**	**−**	**−**		**−**	**−**		**+**	**−**
CIRRHOSIS AND LIVER TRANSPLANT RECIPIENTS											
Becatti et al. [102] (2020)	Fibrinogen from cirrhosis patients	**−**	**+**	**−**	**−**	CD-Dityr-IF-PC	**−**	**−**	**−**	**+**	**−**
Gligorijević et al. [131] (2018)	Plasma from cirrhosis patients					PC-CD-IF					
Gitto et al. [132] (2024)	Fibrinogen from liver transplant recipients	**−**	**+**	**−**	**−**	CD-Dityr-IF	**−**	**−**	**−**	**+**	**−**
Hugenholtz et al. [133] (2016)	Plasma from cirrhosis patients	**−**		**=**		PC	**=**		**−**	**=**	
TRAUMA											
White et al. [134] (2016)	Plasma from trauma patients	**−**						**−**			**+**
CANCER											
Posch et al. [53] (2021)	Plasma from patients with newly diagnosed or recurrent cancer	**+**									**−**
Nowak et al. [135] (2017)	Plasma from MM patients		**+**	**=**	**=**	PC					**+**
Ząbczyk et al. [136] (2019)	Plasma from patients with lung cancer	**+**					**−**		**−**	**+**	**−**
Ullah et al. [137] (2024)	Plasma from patients with peritoneal cancer patients	**+**	**−**					**+**			**−**
Gronostaj et al. [138] (2013)	Plasma from patients with digestive tract cancers		**−**	**=**					**−**		**−**
Małecki et al. [139] (2015)	Plasma from essential thrombocythemia patients			**+**					**−**		**−**
Undas et al. [140] (2014)	Plasma from MM patients		**+**	**−**					**−**	**+**	**−**
Mrozinska et al. [141] (2019)	Plasma from patients after unprovoked venous thromboembolism with malignancy diagnosed during follow-up								**−**		**−**
de Waal et al. [142] (2020)	Plasma from colorectal cancer patients	**+**					**+**			**+**	
Goncalves et al. [143] (2021)	Plasma from breast cancer patients	**=**								**+**	
Ghansah et al. [144] (2024)	Plasma from MM and MGUS patients	**+**									**−**
Bønløkke et al. [145] (2023)	Plasma from patients with lymphoma										**−**
INFECTIONS											
Okazaki et al. [146] (2024)	Plasma from severe COVID-19 patients	**+**									**−**
Davies et al. [147] (2018)	Plasma from septic shock patients										**−**
Hammer et al. [148] (2021)	Plasma from COVID-19 patients										**−**
Larsen et al. [149] (2021)	Plasma from septic shock patients										**−**
Watson et al. [150] (2022)	Plasma from severe COVID-19 patients										**−**
ALZHEIMER’S DISEASE											
Choi et al. [151] (2002)	Plasma from Alzheimer’s disease patients					PC					
ALZHEIMER’S DISEASE											
Zamolodchikov et al. [152] (2012)	Plasminogen-free Fg + amyloid peptides						**−**			**+**	**−**
Cortes-Canteli et al. [153] (2010)	Transgenic mouse models of AD and plasma from healthy donors + Aβ42 peptide	**+**									**−**
Paul et al. [154] (2007)	Transgenic mouse models of AD										**−**

The table summarizes findings from multiple *ex vivo* studies analyzing the effects of oxidative PTMs on fibrinogen. “+” denotes an increase, “−” denotes a decrease, “=“ denotes no change. PC, protein carbonyls; CD, circular dichroism; IF, intrinsic fluorescence; Dityr, dityrosine crosslinking.

## Abbreviations

The following abbreviations are used in this manuscript:

AAs	amino acids
AD	Alzheimer’s disease
CD	circular dichroism
CV	cardiovascular
diTyr	diTyrosine
ESRD	end-stage renal disease
FPA	fibrinopeptide A
FPB	fibrinopeptide B
FXIII	Factor XIII
GCA	Giant Cell Arteritis
MGUS	monoclonal gammopathy of undetermined significance
MI	myocardial infarction
MM	multiple myeloma
PC	protein carbonyl
Phe	Phenylalanine
PTMs	post-translational modifications
RA	Rheumatoid Arthritis
ROS	reactive oxygen species
TAFI	Thrombin Activatable Fibrinolysis Inhibitor
Trp	Tryptophan
Tyr	Tyrosine
T2DM	type 2 diabetes mellitus
